# Impact of Lactic Acid Bacteria Fermentation on (Poly)Phenolic Profile and In Vitro Antioxidant and Anti-Inflammatory Properties of Herbal Infusions

**DOI:** 10.3390/antiox13050562

**Published:** 2024-05-02

**Authors:** Tarik Ozturk, María Ángeles Ávila-Gálvez, Sylvie Mercier, Fernando Vallejo, Alexis Bred, Didier Fraisse, Christine Morand, Ebru Pelvan, Laurent-Emmanuel Monfoulet, Antonio González-Sarrías

**Affiliations:** 1 Life Sciences, TÜBİTAK Marmara Research Center, P.O. Box 21, 41470 Gebze-Kocaeli, Türkiye; tarik.ozturk@tubitak.gov.tr (T.O.); ebru.pelvan@tubitak.gov.tr (E.P.); 2 Laboratory of Food and Health, Research Group on Quality, Safety and Bioactivity of Plant Foods, Department of Food Science and Technology, CEBAS-CSIC, Campus de Espinardo, P.O. Box 164, 30100 Murcia, Spain; mavila@cebas.csic.es (M.Á.Á.-G.); fvallejo@cebas.csic.es (F.V.); 3 Université Clermont Auvergne, INRAE, UNH, F-63000 Clermont-Ferrand, France; sylvie.mercier@inrae.fr (S.M.); alexis.bred@uca.fr (A.B.); didier.fraisse@uca.fr (D.F.); christine.morand@inrae.fr (C.M.)

**Keywords:** polyphenols, thyme, rosemary, pomegranate, echinacea, CCD18-Co, inflammation, antioxidant

## Abstract

Recently, the development of functional beverages has been enhanced to promote health and nutritional well-being. Thus, the fermentation of plant foods with lactic acid bacteria can enhance their antioxidant capacity and others like anti-inflammatory activity, which may depend on the variations in the total content and profile of (poly)phenols. The present study aimed to investigate the impact of fermentation with two strains of *Lactiplantibacillus plantarum* of several herbal infusions from thyme, rosemary, echinacea, and pomegranate peel on the (poly)phenolic composition and whether lacto-fermentation can contribute to enhance their in vitro antioxidant and anti-inflammatory effects on human colon myofibroblast CCD18-Co cells. HPLC-MS/MS analyses revealed that fermentation increased the content of the phenolics present in all herbal infusions. In vitro analyses indicated that pomegranate infusion showed higher antioxidant and anti-inflammatory effects, followed by thyme, echinacea, and rosemary, based on the total phenolic content. After fermentation, despite increasing the content of phenolics, the antioxidant and anti-inflammatory effects via reduction pro-inflammatory markers (IL-6, IL-8 and PGE_2_) were similar to those of their corresponding non-fermented infusions, with the exception of a greater reduction in lacto-fermented thyme. Overall, the findings suggest that the consumption of lacto-fermented herbal infusions could be beneficial in alleviating intestinal inflammatory disorders.

## 1. Introduction

In recent decades, consumers’ increasing choice and consumption of nutritionally enriched and health-promoting foods has sparked global interest in the development of fermented functional foods [1]. Thus, although since ancient times, humans have consumed and produced foods and beverages that have been subjected to fermentation, such as dairy products, beer, and bread, the use of fermentative starters is becoming an aspect of growing interest in the field of food biotechnology to develop new functional foods and beverages [2]. Briefly, the fermentation method in foods and beverages is based on the action of microorganisms, which induce the conversion of food components by microbial enzymes, causing desirable biochemical changes that can provide many benefits to foods extending their shelf-life, nutritional value, and sensory properties and providing many beneficial components for health [2,3]. In this sense, fermented foods may enhance health benefits, including antioxidant, anti-inflammatory, anti-allergenic, anti-microbial, and anti-diabetic effects, among others. This is boosted by the potential probiotic effects of their constituent microorganisms, if they are still present, as well as by the enzymatic bioconversion products to biologically active metabolites (i.e., exopolysaccharides, vitamins, minerals, phenolic compounds, bioactive peptides, organic acids, free amino acids, etc.) [2,3,4,5]. Most of the functional foodstuffs are produced by species and (or) strains belonging to lactic acid bacteria (LAB), which are generally recognized as safe (GRAS) and confer a multitude of functional and sensory properties [6,7]. In this regard, for outside plant-based fermented foods or drinks which are currently widely studied, the LAB starter cultures are directed towards the fermentation of novel substrates from plant foods, herbs, or spices in order to improve their health-promoting effects by stimulating the release or production of bioactive metabolites [8,9,10].

Herbs or plants parts such as roots, leaves, or flowers have been traditionally used to prevent illness, maintain health, and cure some diseases, and many of these have been used ubiquitously to prepare herbal infusions or teas. Herbal teas or infusions commonly refer to “non-*Camellia sinensis* derived infusions”, prepared from boiling fresh or dried parts of edible plants. These beverages are becoming increasingly popular worldwide due to their diverse taste, caffeine-free nature, and potential beneficial effects attributed mainly to being rich in multiple bioactive compounds [11,12]. In general, herbal teas have attracted the interest of researchers due to their beneficial properties, such as antioxidant, anti-inflammatory, anti-microbial, and anti-cardiovascular-disease effects, among others. Most of these health-related effects have been attributed to their high content in phytochemicals, such as (poly)phenols [11,12,13,14,15,16]. Among various herbal teas, thyme, rosemary, echinacea, and pomegranate peel are often chosen for their appealing flavors and potential health benefits, including antioxidant and anti-inflammatory properties, which may be attributed to their phenolic compounds. Thus, rosmarinic acid and luteolin derivatives are the main (poly)phenols in rosemary and thyme [17,18], while ellagitannins and ellagic acid are the primary phenolics in pomegranate peel [19]. Additionally, chicoric and caftaric acids are the most abundant in echinacea extracts [20,21]. However, most of these phenolic compounds are poorly absorbed and usually occur as glycosides, considered biologically inactive, and their bioavailability requires the initial hydrolysis of the sugar moiety by intestinal β-glucosidases producing their aglycones. In addition, they can undergo bioconversion mediated by gut microbiota, in which they are broken down into smaller molecules via enzymatic reactions to facilitate their bioavailability and biological activity in both the gastrointestinal tract and systemic tissues [22]. Therefore, considering these limitations, the fermentative biotransformation approach using LAB may be an effective strategy to improve the bioaccessibility, bioavailability, and bioactivity of phenolic compounds present in herbal infusions.

The objective of this study was to evaluate, for the first time, the effect of the lacto-fermentation on the phenolic profile of four herbal infusions produced from two aromatic herbs (thyme and rosemary) and two plant extracts (*Echinacea pupurea* flower extract and pomegranate peel extract) rich in phenolic compounds in order to elucidate whether lacto-fermentation of these infusions can contribute to enhance their antioxidant and anti-inflammatory activities. To obtain lacto-fermented herbal teas, different strains of *Lactiplantibacillus plantarum* were used. Subsequently, the phenolic profile of each herbal infusion was evaluated by analyzing it before and after lactic fermentation. The in vitro antioxidant effects and their anti-inflammatory effects on human colon fibroblasts (CCD18-Co), which induced an inflammatory cytokine, were also explored. Our results provide a scientific basis for highlighting the effective bioactivity of lacto-fermented herbal infusions.

## 2. Materials and Methods

### 2.1. Reagents

Analytical HPLC-grade chemicals, including acetonitrile (ACN), formic acid, dimethyl sulfoxide (DMSO), and methanol (MeOH), were obtained from J.T. Baker (Deventer, The Netherlands). BMS-345541 was purchased from Selleck (Houston, TX, USA). Ethanol (≥99.8%), and Man, Rogosa, and Sharpe (MRS) broth were purchased from Merck (Darmstadt, Germany). Gallic acid was purchased from Extrasynthese (Genay, France). All other reagents were purchased from Sigma-Aldrich (St. Louis, MO, USA), unless stated otherwise. Ultrapure Millipore water was used throughout the study, generated by a Milli-Q water (18.2 MmΩ) device (Merck, Darmstadt, Germany).

### 2.2. Plant Materials and Preparation of Extracts

Pomegranate peel (*Punica granatum* L.) was purchased from a local supplier (Doğan Baharatçılık Kimyevi Maddeler Tic. ve San. A.Ş., İstanbul, Türkiye). Whole aerial parts of thyme (*Thymus vulgaris*) were purchased from a local market (produced by Sanita Tarım Ürünleri Baharat, Kozmetik San.Tic.A.Ş., İzmir, Türkiye). Rosemary (*Rosmarinus officinalis* L.) leaves were obtained from BATEM (Republic of Türkiye Ministry of Agriculture and Forestry West Mediterranean Agricultural Research Institute). Echinacea (*Echinacea purpurea* L.) flower extract was obtained from a local producer (HMC Naturel Tarım ve Hayvancılık San. Tic. Ltd. Şti., Dodurga, Çorum, Türkiye).

Thyme plant materials were directly used without grinding before fermentation. Pomegranate peel was ground before fermentation, as described below. Rosemary leaves were extracted with 80% ethanol, which was subsequently evaporated. The remaining aqueous phase was lyophilized (Christ Epsilon 2–4 Lyo-Screen-Control (LSC), Osterode am Harz, Germany) to obtain a dry powder used for fermentation. Echinacea flowers were extracted with MilliQ water using a pilot-scale continuous counter current extractor (Niro Atomizer, AC-27, Soeborg, Denmark) and spray-dried (Minor Spray Dryer, Niro Atomizer, Soeborg, Denmark) to obtain a powder for further fermentation.

### 2.3. Strains Descriptions

Two different strains of lactic acid bacteria were isolated from local plant sources in Türkiye by TUBİTAK MAM and were identified by 16S rDNA sequencing. Strain A, identified as the *Lactiplantibacillus plantarum* 129 J1 strain, was isolated from olive and developed through an evolutionary engineering strategy, where superior strains were selected among mutant populations by the gradual application of selective pressure to mimic the natural evolutionary process. The resulting strain, J1, was able to almost completely survive passage through the upper gastrointestinal tract. Strain B, identified as the *Lactiplantibacillus plantarum* P1 strain, was isolated from fermented traditional black carrot juice, and was selected due to its ability to decrease pH rapidly and suppress competitive flora.

### 2.4. Fermented-Infusions Preparation

Firstly, 50 g of thyme or pomegranate peel, as detailed above, was dissolved in water at a ratio of 1:20 (*w*/*v*) and incubated at 90 °C for 15 min. The rosemary and echinacea were dissolved in water at a ratio of 1:50 (*w*/*v*) and incubated at 90 °C for 1 min. Next, for all beverages, 500 mL of MilliQ water at room temperature was added to the samples, and the bottles were cooled at 30 °C. The prepared beverages were inoculated a rate of 1% of each strain grown for 48 h at 30 °C. The pH was measured before and after fermentation to confirm its decrease (from pH values above 5 to values below 4.4, depending on the beverage) as a result of the fermentation process (Supplementary Table S1).

Selected samples were transferred to 50 mL screw-cap polyethylene centrifuge tubes and centrifuged (Hettich Rotina 420R, Sérézin du Rhône, France) at 3500× *g* for 2 min to clarify the extracts. The supernatants were transferred to new tubes and pasteurized in a water bath at 85 °C for 10 min. Next, the samples were aseptically stored at −80 °C for further analyses.

### 2.5. Determination of Total Phenolic Content

#### Analysis of Polyphenolic Content (Total) and by HPLC-MS/MS

The total phenolic content (TPC) of herbal infusions was determined using a previously reported method with minor modifications [23]. Briefly, infusion samples were diluted twice in purified water, and 2 mL of these diluted solutions were mixed with 1 mL of undiluted Folin–Ciocalteu reagent. The volume was finally adjusted to 25 mL with a sodium carbonate solution (150 g/L). After incubation for 30 min at room temperature, absorbance was recorded at 740 nm using a Jasco V-630 spectrophotometer (Lisses, France), and the result was expressed in mg of gallic acid equivalents (GAE) per g of dry material using a standard curve of gallic acid (0.005–0.1 mg/mL).

### 2.6. HPLC-MS/MS Analysis

The phenolic content of plant material (raw extracts) from echinacea, thyme, rosemary, and pomegranate peel was analyzed as described elsewhere [24]. Briefly, 50 mg of each sample was dissolved in a solution containing 10 mL of MeOH/DMSO/H_2_O (40:40:20, *v*/*v*/*v*) supplemented with 0.1% HCl (*v*/*v*). The samples were then vortexed for 2 min, subjected to ultrasonic bath sonication for 5 min, and centrifuged at 4000× *g* for 5 min at room temperature. This extraction process was repeated using a 5 mL extraction solution to maximize the phenolic compound yield. Finally, the resulting supernatant of all extracts was filtered through a 0.45 µm polyvinylidene difluoride (PVDF) filter prior to HPLC-MS/MS analysis. Each sample underwent extraction and analysis in triplicate to ensure consistency.

On the other hand, for the analysis of the phenolic profile of non-fermented and lacto-fermented beverages, 1 mL was extracted with MeOH in a 1:1 (*v*/*v*) proportion to remove contaminants from the fermentation process. The samples were homogenized using a vortex for 2 min and centrifuged at 10000× *g* for 15 min, and the supernatant was recovered. Next, each sample was evaporated in a speed vacuum and re-suspended in 150 µL of MeOH. The final samples in MeOH were filtered using 0.45 µm PVDF filters and transferred to vials before HPLC analysis.

HPLC analyses were performed on an Agilent 1200 HPLC system with a photodiode array detector (DAD) (Agilent Technologies, Waldbronn, Germany) and an ion trap (IT) mass spectrometer detector in series (Bruker Daltonik, Bremen, Germany). A reverse-phase Poroshell 120 C18 column (100 × 3.0 mm, 2.7 µm) was utilized. The mobile phases consisted of water:formic acid (99:1) as A and acetonitrile (ACN) as B, with a flow rate of 0.6 mL/min. The gradient was as follows: 0–1% B at 0 min, 1–40% B at 0–20 min, 40–90% B at 20–30 min, and 90% B at 30–33 min, followed by a return to initial conditions (1% B) in 1 min with column re-equilibration for 5 min. The injection volume was 12 µL. UV-Vis spectra were acquired in the range of 200 to 600 nm. In the mass spectrometer, nitrogen served as the drying and nebulizing gas, with a pressure of 65 psi, flow of 11 L/mL, and temperature of 350 °C.

The identification of compounds was performed by taking information about their elution order; UV spectra; molecular weight; fragmentation by MS/MS; and, whenever possible, chromatographic comparison with authentic standards. External calibration curves with appropriate standards belonging to the different families of phenolic compounds were used for the quantification. Rosmarinic acid from thyme and rosemary extracts was quantified with its own standard at 320 nm. Punicalin and punicalagins from pomegranate were quantified with their own standards, and ellagic acid (and derivatives) with ellagic acid, at 360 nm. Flavanones were quantified at 340 nm with hesperidin and eriocitrin, flavones at 340 nm with apigenin, and flavonols at 360 nm with quercetin. The hydroxycinnamic acids in echinacea extract were quantified at 320 nm with chicoric acid or caffeic acid.

### 2.7. Antioxidant Activity

The DPPH (2,2-diphenyl-1-picrylhydrazyl) scavenging activity was evaluated according to a previously published protocol [25]. Briefly, 50 µL of reference and digestive solutions were mixed with 2.5 mL of the fresh radical mixture (25 µg/mL in MeOH). After incubation at room temperature for 30 min, the absorbance was measured at 515 nm. The DPPH scavenging capacity was expressed as micromoles of trolox (6-hydroxy-2,5,7,8-tetramethyl-3,4-dihydrochromene-2-carboxylic acid) equivalent (µmol TE) per gram of dry material using a standard curve of Trolox (100–3000 µmol/L).

The ferric reducing antioxidant power (FRAP) assay was performed according to Katalinić et al. [26] with minor modifications. Stock solutions included a 300 mM acetate buffer (3.1 g of C_2_H_3_NaO_2_·3H_2_O and 16 mL of C_2_H_4_O_2_) pH 3.6, a 10 mM TPTZ (2,4,6-tripyridyl-s-triazine) in 40 mM HCl solution, and a 20 mM FeCl_3_·6H_2_O solution. A fresh FRAP working solution was then prepared by mixing 25 mL of acetate buffer, 2.5 mL of TPTZ solution, and 2.5 mL of FeCl_3_·6H_2_O solution and kept at 37 °C before use. Next, 10 µL of each sample was added to react with 200 µL of the FRAP solution. After 30 min at room temperature in the dark, the absorbance of the ferrous colored product was recorded at 593 nm. Results were expressed in µmol TE/g of sample using a standard trolox curve (50–750 µmol/L).

### 2.8. Xanthine Oxidase (XO) Inhibitory Activity

Inhibition of XO activity was determined according to the method described by Sowndhararajan et al. [27], with slight modifications. Reference and digestion samples (70 µL) were incubated in the dark at 25 °C with 120 µL of PBS (phosphate buffer solution), 120 mM pH 7.5, and 100 µL of a 1.5 mM xanthine solution. After 5 min, 10 µL of XO 0.30 UI/mL was added. The progress of the reaction (uric acid production) was measured at 293 nm, and the percentage of inhibition was calculated. Six sample concentrations (0.58–2.33 mg/mL) were used for analysis, and activities were expressed as IC_50_ in mg of dry material/mL.

### 2.9. Cell Culture

Myofibroblasts of the colon CCD18-Co cell line were obtained from the American Type Culture Collection (ATCC, Rockville, MD, USA). CCD18-Co cells were maintained in Eagle’s minimum essential medium (EMEM) supplemented with 10% fetal bovine serum (FBS), antibiotics (streptomycin and penicillin at 100 mg/mL and 100 U/mL, respectively), 1.5 g/L sodium bicarbonate, 1 mM sodium pyruvate, 0.1 mM nonessential amino acids, and 2 mM L-glutamine. Cells were maintained at 37 °C in the presence of 5% CO_2_. The range of population doubling levels (PDL) used in all experiments was from 26 to 32.

### 2.10. Cell Viability and Inflammatory Assay

CCD18-Co cells were subcultured at 2000 cells per well on 96-well plates and incubated with media as described above for 1 day. To select the highest nontoxic concentrations of non-fermented and fermented from each herbal infusion, the osmolarity and pH of a range of percentages (5, 2.5, 1 and 0.5%) were evaluated using a vapor pressure osmometer 5520 (VAPRO Wescor, Logan Utah, UT, USA) and a pH indicator paper (Neutralit, pH 5.5-9.0, Merck), respectively. Additionally, to confirm that the treatments did not exert an antiproliferative and/or cytotoxic effect, the CCD18-Co cell viability and proliferation were measured using the MTT reduction assay at 24 h, as described by González-Sarrías et al. [28]. Once these parameters were optimized, the attached cells in 96-well plates were incubated in EMEM supplemented with 0.1% FBS (*v*/*v*) for 24 h. Then, cells were treated with the sterilized (filtered by 0.22 μm) non-fermented and fermented samples at non-cytotoxic concentrations (2.5%) and co-treated with 1 ng/mL IL-1β for 16 h. Cells in the absence of IL-1β were used as negative controls (CT). BMS-345541 (BMS) at 5 µM was assayed as a positive control of the anti-inflammatory effect. After the inflammatory assay, the culture medium was collected and frozen at −80 °C for further analysis. Although doses of 2.5% obtained non-statistical differences to control cells of cell viability and proliferation values, the cell proliferation data obtained using an MTT reduction assay after each treatment were used to normalize the values of inflammatory markers. Assays were repeated three times (n = 3), with 6 measurements within each replicate.

### 2.11. Effect of the Lacto-Fermented Beverages on Cytokine Production and PGE2 Biosynthesis in IL-1β-Stimulated Cells

Pro-inflammatory cytokines, including IL-8 and IL-6, were measured in culture medium using their corresponding ELISA kits from PeproTech (Rocky Hill, NJ, USA), and the absorbance at 405 and 650 nm (reference wavelength) was detected using a microplate reader (Infinite M1000 Pro, Tecan, Grodig, Austria). The analysis of PGE2 in the culture medium was measured using an ELISA kit from Cayman (San Diego, CA, USA) and the same absorbance-detecting microplate reader. The data, expressed as average ± SD, were the results of three independent biological replicates (n = 3). The culture medium of the different treatments (carried out in each replicate) were pooled from six to eight different wells.

### 2.12. Statistical Analysis

All experimental data were expressed as the mean ± standard deviation (S.D.). The two-tailed unpaired Student’s *t* test was used for statistical analysis of the data using SPSS Software, version 27.0 (SPSS Inc., Chicago, IL, USA) or Prism 6, version 6.01 (GraphPad). Graphs were constructed using GraphPad Prism 9.1.1 software (GraphPad Software, San Diego, CA, USA). To examine the correlation between phenolic profiles and antioxidant and anti-inflammatory effects, Pearson correlation analysis using MetaboAnalyst 6.0. was performed. The difference was considered to be statistically significant at a *p* value < 0.05.

## 3. Results

### 3.1. Screening of Lacto-Fermented Beverages Based on pH

From a regulatory point of view, for measuring the impact of food safety criteria on public health, foods are classified into low- and high-acid foods according to their pH. Foods lower than pH = 4.6 are considered as high-acid ones, since the spores of an extremely dangerous microorganism called *Clostridium botulinum* cannot germinate or produce toxins below this pH range, even if the food is pasteurized [29]. For this reason, a first screening measuring the pH value as an indicator for preservation and safety was carried out after the fermentation process (with each strain A or B) for each plant material. Thus, all lacto-fermented beverages showed pH values low enough to be considered as high-acid foods, and, therefore, safe without the need to add additives (Supplementary Table S1). Next, they were pasteurized and collected for further analyses.

### 3.2. Phenolic Characterization of Plant Material

A comprehensive HPLC-MS/MS analysis of the different plant material used for the lacto-fermentation of thyme, pomegranate, rosemary, and echinacea beverages is detailed in Supplementary Table S2. All phenolic compounds were identified using a comprehensive analytical approach, which involved assessing their retention time, mass, mass fragmentation patterns, and UV-Vis characteristics. To validate these identifications, comparisons were made with authentic standards whenever feasible. In thyme, the predominant compounds identified were chrysoeriol glucoside, followed by eriodictyol and rosmarinic acid. In pomegranate peel extract, punicalagin and ellagic acid emerged as the most abundant phenolic compounds detected. In the case of rosemary, the principal compounds observed were the flavonol isorhamnetin-3-*O*-glucoside and the flavone luteolin glucoside. Lastly, the echinacea extract exhibited chicoric acid and caftaric acid as the most prevalent compounds (Supplementary Table S2).

### 3.3. Phenolic Profile Comparison of Non-Fermented and Lacto-Fermented Beverages

The total (poly)phenol content (TPC) and the phenolic profile of both fermented and non-fermented infusions were determined (Table 1 and Table 2, respectively). Both the TPC and the phenolic profile differed for the four infused beverages. For TPC assessed using the Folin–Ciocalteu assay, lactic acid fermentation with strain A and B resulted in a significant higher TPC (from 4% to 36% increase) compared to the non-fermented infusions (Table 1). The highest effect was observed after fermentation for thyme infusion. On the other hand, both fermentations slightly modulated the TPC of pomegranate and echinacea infusion, but not rosemary infusions. Furthermore, no significant difference was observed for TPC after fermentation between strain A and strain B.

Regarding the (poly)phenol profiles obtained by HPLC-MS/MS, as depicited in Table 2, both strain A and B exhibited increases in most of the quantified phenolics, albeit showing some differences between strains. Specifically, in thyme, differences were observed in certain phenolics, such as eriodictyol, quercetin, and salvianolic acid A. Regarding rosemary, differences were noted in isorhamnetin-3-glucoside, rosmarinic acid, and luteolin glucoside; in echinacea, all phenolics showed significant differences between strain A and B, except for caffeic acid and chlorogenic acid; and in pomegranate herbal tea, only punicalin showed a notable difference.

In both thyme-based fermented infusions, infusion phenolics such as luteolin, chrysoeriol, erodictyol, rosmarinic acid, quercetin, and salvianolic acid were quantified in greater amounts compared to the non-fermented one. Chrysoeriol glucoside exhibited the most pronounced increase with strain B, while eriodictyol was the only compound that did not show any significant increase compared to the non-fermented thyme herbal infusion. Interestingly, rosmarinic acid, the main compound found in non-fermented thyme, exhibited 1.4 and 1.6 times higher concentrations after fermentation with strains A and B, respectively, compared to the concentrations detected in the non-fermented beverage.

In regard to the phenolics detected in the rosemary infusion, rosmarinic acid exhibited a 2.5-fold increase with strain A and a 4.9-fold increase when it was fermented with strain B compared to the non-fermented beverage. Additionally, the difference between strains A and B was statistically significant (Table 2). Another pronounced increase was observed with hesperidin, showing a 14.5-fold increase with strain A and a 4.5-fold increase with strain B compared to the non-fermented rosemary beverage.

Surprisingly, in the case of echinacea infusions, a higher pronounced effect was observed mainly after fermentation with strain A. Thus, although caftaric acid, chicoric acid, feruloylcaffeoyltartaric acids, and neochlorogenic acid concentrations increased after lacto-fermentation with both strains compared to the non-femented sample, the use of strain A resulted in the most notable increase in all phenolic compounds.

Finally, for the herbal infusion from pomegranate peel, it is noteworthy that only ellagic acid hexose, ellagic acid, and granitin B exhibited increased concentrations following lacto-fermentation with both strain A and B in association with new compounds such as gallagic acid and ellagic acid-pentose. Conversely, punicalin demonstrated a significant decrease in its concentration with lacto-fermentation using both strains.

### 3.4. Antioxidant Capacity

The antioxidative effect of the four lacto-fermented infusions and their corresponding non-fermented ones was tested by measuring the in vitro DPPH activity, FRAP activity, and xanthine oxidase inhibition (XO) (Table 3). Thyme-fermented, rosemary-fermented, and echinacea-fermented infusions exhibited significant higher DPPH activity than their respective non-fermented infusions. The FRAP activity of thyme infusions was slightly improved by the fermentation. Regarding rosemary-fermented and echinacea-fermented infusions, they showed significantly increased FRAP activity compared to the non-fermented rosemary infusion and non-fermented echinacea infusion, respectively. Fermentation did not modulate the inhibition of XO of pomegranate and echinacea infusions, but significantly improved it in thyme and rosemary beverages after both fermentations.

### 3.5. Effects on Cell Viability in CCD18-Co Myofibroblasts

After the evaluation of cell viability, as well as the osmolarity and pH, using the MTT assay, the highest non-cytotoxic dose of both fermented and non-fermented infusions was selected to run the in vitro colonic inflammatory model. The selected concentration for each treatment of 2.5% in culture medium afforded pH values of 7 and osmolality values of 285–305 mmol/Kg, which are within the tolerance limits of this human cell model and showed no statistically significant differences on cell viability (over 95%) compared to untreated CCD18-Co cells.

### 3.6. Effect on IL-1β-Induced IL-6, IL-8 and PGE2 Production in CCD18-Co Myofibroblasts

**Figure 1:**
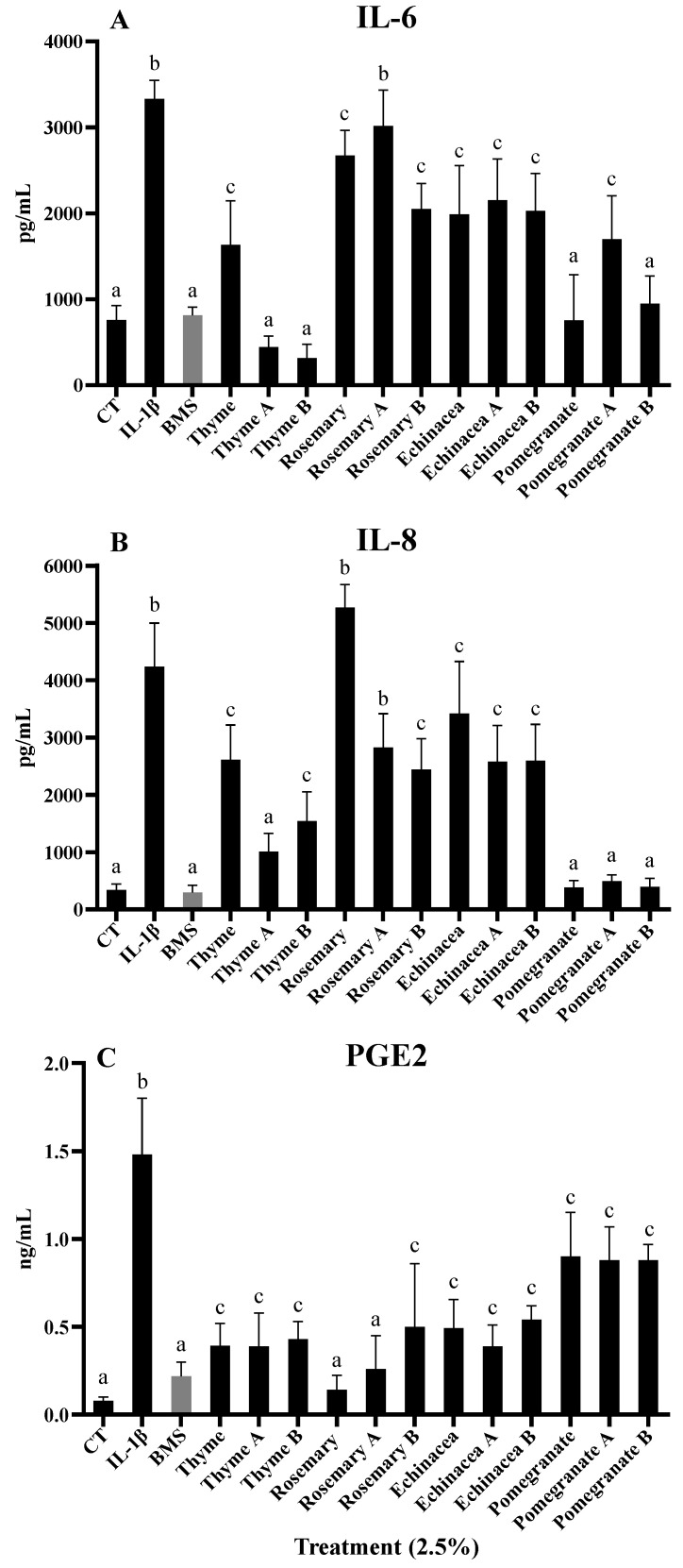
Pro-inflammatory cytokines (IL-6 (**A**) and IL-8 (**B**)) and PGE_2_ levels (**C**) produced in the CCD18-Co culture media after 18 h of treatment, as measured by ELISA after exposure to IL-1β (1 ng/mL) alone or in combination with the four lacto-fermented herbal teas and their corresponding non-fermented beverage samples at a subtoxic dose of 2.5%. The selective IKK-2 inhibitor (BMS 345541; BMS) at 5 μM was assayed as a positive control of the anti-inflammatory effect. Results are shown as the mean ± SD of three independent experiments. Different letters indicate significant differences *p* < 0.05.

The anti-inflammatory effect of the four lacto-fermented beverages and their corresponding non-fermented beverage samples at the subtoxic dose of 2.5% on IL-1β-induced CCD18-Co myofibroblasts was tested by measuring the IL-6, IL-8, and PGE2 production for 18 h. The exposure of the cells to IL-1β led to an increase (*p* < 0.05) in the release of both pro-inflammatory cytokines and PGE2 compared to both untreated samples (CT) (Figure 1). The inflamed cells co-treated with non-fermented beverages samples of each plant material extract showed a reduction (*p* < 0.05) in the concentration of inflammatory markers, with the exception of rosemary for the IL-8 levels. Among the treatments, pomegranate peel beverage showed higher reductions for IL-6 and IL-8, followed by thyme, echinacea and rosemary beverages, respectively (Figure 1A,B). On the contrary, regarding the PGE2 production, the treatment with non-fermented beverages of rosemary and thyme showed the highest reduction (Figure 1C). Regarding the lacto-fermented beverages with strain A or B, the reduction in IL-6 levels was similar or even lower than of their corresponding non-fermented beverages, but still significant compared to untreated inflamed cells (*p* < 0.05). An exception was a greater reduction for both lacto-fermented thyme beverages compared to their corresponding non-fermented ones (*p* < 0.05) (Figure 1A). Regarding IL-8 values, lower but statistically significant (*p* < 0.05) values were detected for lacto-fermented thyme (using Strain A) and rosemary (using Strain B) beverages, but not for the other samples compared to their corresponding non-fermented beverages, although a non-significant trend was also observed for both lacto-fermented echinacea beverages and rosemary fermented with Strain A (Figure 1B). Finally, no statistically significant differences were observed in the reduction in PGE2 levels between the four lacto-fermented beverages and their corresponding non-fermented ones (Figure 1C).

### 3.7. Correlation of (Poly)Phenolics with the Antioxidant and Anti-Inflammatory Effects

**Figure 2:**
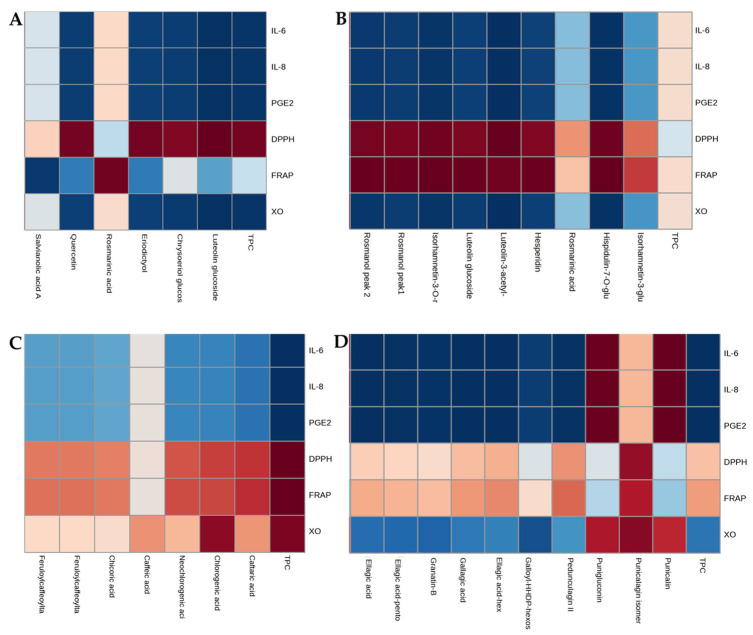
Heatmap analysis of the Pearson correlation between the total phenolic content (TPC) and individual (poly)phenolics detected with the three pro-inflammatory markers (IL-6, IL-8, and PGE_2_) and antioxidant activities measured by DPPH, FRAP, and XO inhibition. Thyme (**A**), rosemary (**B**), echinacea (**C**), and pomegranate peel (**D**) infusions. Values higher than the averages (positive correlation) are indicated in red, and lower values in blue (negative correlation).

The correlation between the total polyphenol content (TPC) and individual (poly)phenol detected in each of the herbal infusions was analyzed, including data of the non-fermented infusions and those after two fermentations. The antioxidant activity (measured by DPPH and FRAP, and inhibition of XO) and the three pro-inflammatory markers (IL-6, IL-8, and PGE2) were also analyzed. Correlation and *p* values for all analyses in each herbal infusion are detailed in Supplementary Tables S3–S6. According to the data represented in Figure 2A, the Pearson correlation analysis revealed significantly negative correlations with the TPC of thyme with all pro-inflammatory markers and XO inhibition, while a positive correlation was found with DPPH activity. Individually, luteolin, chysoeriol glucoside, eriodictyol, and quercetin showed similar correlations, while rosmarinic acid only correlated positively with FRAP activity (r = 0.96, *p* = 0.002) (Figure 2A). For rosemary infusions, no statistically significant correlations were found with TPC for any marker evaluated. However, our analysis revealed significantly negative correlations with all individual (poly)phenols detected, except for rosmarinic acid, with all pro-inflammatory markers and XO inhibition. In addition, positive correlations were found with DPPH and FRAP activity (Figure 2B). Regarding echinacea infusions, the Pearson correlation analyses revealed significantly negative correlations between TPC with all pro-inflammatory markers and XO inhibition, while positive correlations were found between DPPH and FRAP activity. However, a positive correlation was also found for XO inhibition. Individually, a similar trend, although not statistically significant, was found for all phenolics detected in echinacea, with the exception of caffeic acid (Figure 2C). Finally, for pomegranate peel infusions, statistically significant inverse correlations between TPC and all pro-inflammatory markers were found. However, although a trend was observed, there were no statistically positive correlations with DPPH or FRAP activity nor an inverse correlation with XO inhibition. Among individual (poly)phenolics, most of them showed similar trends to TPC, with the exception of punicalin and punigluconin, which showed positive correlations with the three pro-inflammatory markers (Figure 2D).

## 4. Discussion

The consumption of and demand for herbal teas and infusions continues to increase globally as caffeine-free beverages with attractive flavors, as well as many functional health benefits. Several aromatic herbs and plant extracts have traditionally been used for their antioxidant properties and for treating intestinal inflammation, indigestion, infections, etc., effects which are attributed to the predominant (poly)phenol compounds in these plant-based products [11,14,15]. However, although these bioactive compounds could be ingested in significant amounts, most (poly)phenols present in plant extracts have low bioavailability and reach the colon almost unaltered, where they can be metabolized by the gut microbiota to release numerous metabolites that are then absorbed and display biological effects [22,30]. In general, the biological action of (poly)phenols depend upon their bioaccessibility, which refers to the fraction of an ingested compound that is available for absorption in the gut and can also exert health benefits in the intestinal tract. In this line, different factors can affect the bioaccesibility of phenolics present in plant extracts, such as their water solubility, their presence mostly as glycosides, and their tight bond to food matrices [31]. Therefore, in recent decades, in order to enhance the bioaccessibility and bioavailability of the plant-food (poly)phenols and, therefore, their health benefits, different technological and biotechnological processes have been developed, including fermentation with lactic acid bacteria (LAB), which could be able to release phenolic compounds from the matrix and (or) lead to modifications and conversion to other phenolic compounds with improved bioavailability [8,31,32]. Herein, the present study has corroborated the impact of lacto-fermentation on several herbal infusions from thyme, rosemary, echinacea, and pomegranate peel on the (poly)phenolic composition and the improvement of their in vitro antioxidant and anti-inflammatory effects compared to their corresponding non-fermented infusions. The total (poly)phenol content (TPC) and the phenolic profile analyses by HPLC-MS/MS revealed that lacto-fermentation markedly increased the levels of most (poly)phenolics in all herbal infusions compared to their non-fermented counterparts. This is in agreement with many studies that have reported that LAB species and strains are able to increase the production of polyphenolic compounds in many foods and beverages [4,33,34,35]. Under our specific analysis conditions, we did not detect the formation of new compounds, as we followed a targeted strategy to compare fermented vs. non-fermented infusions. However, despite the significant differences observed between fermented and non-fermented infusions for many compounds, we cannot rule out the possibility of new bioactive compounds (e.g., phenolics) forming during the fermentation process.

In our study, we isolated from olive and black carrot two strains of *Lactiplantibacillus plantarum*, which are commonly used and found in foods, dairy products, and beverages and have the Generally Recognized as Safe (GRAS) status from the US Food and Drug Administration (US FDA) and the Qualified Presumption of Safety (QPS) status from the European Food Safety Authority (EFSA) [36,37]. Among herbal infusions, thyme infusion showed the highest increase in the (poly)phenolic content after fermentation, followed by the echinacea infusion. Surprisingly, although *L. plantarum* possesses β-glucosidase activities, the lacto-fermentation did not decrease, but rather increased the content of phenolic glycosides presents in thyme and rosemary, such as luteolin, chrysoeriol, isorhamnetin, or hispidulin. In contrast, previous studies have reported the high glycosidase activity of *L. plantarum,* with an improvement in the bioaccessibility and bioavailability of food phenolic compounds such as isoflavones and other flavonoid glycosides, such as quercetin or kaempferol. This is accompanied by an increase in their antioxidant activity [34,35,38,39]. However, according to these studies, differences were observed in the deglycosylation rate depending on the strains used (i.e., greater activity for *L. plantarum* 748T than for *L. plantarum* 9567), while in our study, the 16S rDNA sequencing indicated that the strains used for fermentation were *L. plantarum* 129 J1 and P1, which could be present lower β-glucosidase activity than others. Furthermore, another recent study described that some strains of *L. plantarum* do not possess any enzyme exhibiting β-glucosidase activity [40]. Therefore, our study corroborated that the impact of fermentation can be dependent both on LAB species with GRAS status and between strains of the same species. On the other hand, the release of phenolic compounds after lacto-fermentation could be related to the action of other enzymes that contribute to the breakdown of plant cell wall, thus improving their bioaccessibility. In this regard, other microbial enzymes apart from glucosidases, such as amylases, cellulases, chitinases, esterases, invertases, etc., produced during fermentation could degrade the cell walls of the plants and therefore improve the extraction of phenolic compounds [41].

Regarding the health benefits of lacto-fermentation, the antioxidant and anti-inflammatory effects of the lacto-fermented beverages and their corresponding non-fermented infusions were measured. We report here for the first time that thyme-, rosemary-, echinacea-, and pomegranate-based non-fermented infusions exhibit in vitro antioxidant effects similar to other plant-derived products (e.g., extracts or juices) [42,43,44,45]. According to the literature on the health benefits of lacto-fermentation, we observed enhanced antioxidant effects for most of the lacto-fermented infusions. The lacto-fermented thyme and rosemary infusions had an increased radical-scavenging activity (DPPH) and ferric reducing ability (FRAP, as well as to inhibit xanthine oxidase. Similarly, lacto-fermented echinacea infusions showed a significant increase in the DPPH and FRAP activities. Surprisingly, lacto-fermentations had controversial effects on pomegranate infusions, with a decrease in FRAP activity no matter the strain used and a higher DPPH activity when fermented with *L. plantarum* 129 J1 (strain A). These findings are in line with previous studies conducted on echinacea extract fermented with *L. plantarum* 1MR20 [46] and on pomegranate juice fermented with selected *L. plantarum* PU1 [47], which exhibited higher antioxidant effects than their respective unfermented controls. On the other hand, regarding the anti-inflammatory effects, all herbal infusion showed significant decreases in the production of pro-inflammatory markers (IL-6, IL-8, and PGE2) on IL-1β-induced CCD18-Co human colon myofibroblasts. This is in agreement with previous studies conducted in systemic and colon models where the anti-inflammatory effect was attributed to the phenolic fraction [21,48,49,50]. After lacto-fermentation with the two strains of *L. plantarum*, the reduction in all pro-inflammatory markers was preserved, with the exception of a greater reduction for lacto-fermented thyme compared to the corresponding non-fermented one and, to a lesser extent, for the fermented echinacea and rosemary infusions.

Overall, findings suggest that the lacto-fermentation of herbal infusions could enhance their antioxidant and anti-inflammatory effects, and this effect could be explained, at least partly, by the increase in and release of their phenolic fractions. Although no individual phenolics were evaluated, previous preclinical studies have found both antioxidant and anti-inflammatory activities in the gastrointestinal tract for many, such as rosmarinic acid, ellagic acid, chicoric acid, and caffeic acid, which are present in significant amounts in these herbs and plant extracts [51,52,53,54]. Furthermore, in order to explain whether the effect could be mediated by the (poly)phenolics, we ran a Pearson correlation analysis to evaluate a potential statistical correlation between the changes in the (poly)phenolics through lacto-fermentation and the antioxidant and anti-inflammatory effects. Through correlation analysis, we found that both the total phenolic content and most of the detected individual (poly)phenolics were positively correlated with the three evaluated pro-inflammatory markers and the XO inhibition. On the other hand, they were negatively correlated with the antioxidant activities (measured by DPPH and FRAP), mainly for thyme and echinacea, while those of pomegranate peel infusions correlated better with the inflammatory markers.

However, although we obtained some achievements, there are some limitations of the present study, and they should be further investigated. In this line, the antioxidant and anti-inflammatory properties could be also attributed to other fermentation-derived products such as vitamins, bioactive peptides, etc., or the synergy between different bioactives.

Furthermore, although the effect of the fermentation on these herbal infusions clearly improved the release of phenolics, the role of digestion and the interaction with the gut microbiota should be considered. Thus, firstly, during digestion, the structures of the (poly)phenolics can be hydrolyzed and modified, altering their bioavailability and potential health benefits [55]. Therefore, increasing the (poly)phenolic content through lacto-fermentation could preserve its health effects. In this line, a recent study carried out with extracts of different echinacea parts reported that their anti-inflammatory effects were preserved after in vitro gastrointestinal digestion despite a reduction in the concentration of their phenolics in those with higher contents [21]. Regarding the interaction with the microbiota, a higher content of phenolics, included glycosylated forms, which can be cleaved by endogenous or microbial enzymes from gut microbiota to release an absorbable and(or) metabolizable aglycone forms, could favor a higher conversion of phenolic compounds to more biologically active compounds (e.g., ellagic acid to urolithins) [56], as well as the prebiotic effect of the phenolics to positively modulate the gut microbiota [22,57].

## 5. Conclusions

Our results underscore the differential impact of lacto-fermentation on specific phenolic compounds in different herbal infusions, highlighting the complex interplay between microbial strains and phenolic composition during fermentation processes. The results showed that fermentation with *L. plantarum* markedly increased the content (accessibility) of phenolic compounds compared to the non-fermented infusions, with an enhancement of the antioxidant and anti-inflammatory activities. Among the fermented herbal infusions, those from thyme and rosemary showed more significant effects the corresponding non-fermented ones.

In conclusion, this research demonstrates that the health benefits of various herbal infusions can be improved with LAB fermentation based on greater accessibility of their phenolic compounds, thus being able to exert higher antioxidant and anti-inflammatory activities. Overall, this study suggests that lacto-fermentation could be used as tool to produce novel functional herbal infusions or beverages with higher antioxidant and anti-inflammatory activities. Furthermore, our results provide a scientific basis for highlighting the effective bioactivity of lacto-fermented herbal infusions that could be consumed in alleviating certain oxidative-stress-related diseases, intestinal inflammatory conditions, and related disorders.

## Figures and Tables

**Table 1:** Total phenolic content of fermented and non-fermented beverages.

	Non-Fermented	Fermented A *	Fermented B *
**Thyme**	35.15	44.92 (+27.8%)	48.07 (+36.8%)
**Rosemary**	36.11	37.62 (+4.2%)	34.16 (−5.4%)
**Echinacea**	70.09	78.76 (+12.4%)	79.29 (+13.1%)
**Pomegranate peel**	90.01	98.96 (+9.9%)	99.63 (+10.7%)

Values are expressed as mg eq GAE/g of dry matter. * Effect of the lactic acid fermentations on the TPC (two-way ANOVA): fermented (A or B) vs. non-fermented: *p* = 0.0295; fermented A vs. fermented B: *p* > 0.05.

**Table 2:** Comparison of major phenolics detected in fermented and non-fermented beverages.

Herbal Infusion	Phenolic	Non-Fermented	Fermented A	Fermented B
Thyme	Luteolin glucoside	6.19 ± 0.82	19.22 ± 0.97 ^a^	17.45 ± 2.36 ^a^
	Chrysoeriol glucoside	8.72 ± 5.62	42.39 ± 3.69 ^a^	59.15 ± 2.91 ^a,b^
	Eriodictyol	22.30 ± 2.00	27.99 ± 0.85 ^a^	18.89 ± 1.50 ^b^
	Rosmarinic acid ^a^	37.08 ± 3.21	51.40 ± 1.09 ^a^	58.84 ± 2.61 ^a^
	Quercetin ^a^	0.17 ± 0.03	0.63 ± 0.01 ^a^	0.47 ± 0.03 ^a,b^
	Salvianolic acid A	6.37 ± 0.65	12.84 ± 0.39 ^a^	14.80 ± 1.03 ^a,b^
Rosemary	Isorhamnetin-3-glucoside	42.12 ± 4.57	68.66 ± 10.30	43.37 ± 4.64 ^b^
	Hispidulin-7-*O*-glucoside	43.58 ± 1.11	88.48 ± 7.25 ^a^	78.69 ± 3.99 ^a^
	Rosmarinic acid *	19.99 ± 2.60	50.80 ± 1.40 ^a^	97.41 ± 7.42 ^a,b^
	Hesperidin *	2.72 ± 0.47	39.44 ± 1.72 ^a^	25.74 ± 3.61 ^a,b^
	Luteolin-3-acetyl-*O*-glucuronide	4.45 ± 0.60	43.26 ± 0.85 ^a^	48.21 ± 10.58 ^a^
	Luteolin glucoside	1.93 ± 0.21	32.49 ± 0.82 ^a^	21.52 ± 2.93 ^a,b^
	Isorhamnetin-3-*O*-rutinoside	1.71 ± 0.28	29.38 ± 1.48 ^a^	22.31 ± 3.15 ^a^
	Rosmanol peak1	0.36 ± 0.02	3.72 ± 0.10 ^a^	2.55 ± 0.38 ^a^
	Rosmanol peak 2	0.25 ± 0.03	3.61 ± 0.29 ^a^	2.62 ± 0.41 ^a^
Echinacea	Caftaric acid	0.15 ± 0.006	0.58 ± 0.02 ^a^	0.27 ± 0.03 ^a,b^
	Chlorogenic acid	0.03 ± 0.003	0.04 ± 0.01	0.009 ± 0.004
	Neochlorogenic acid	0.02 ± 0.002	0.22 ± 0.03 ^a^	0.05 ± 0.004 ^a,b^
	Caffeic acid *	0.05 ± 0.007	0.30 ± 0.10	0.07 ± 0.01
	Chicoric acid *	1.52 ± 0.08	2.43 ± 0.15 ^a^	1.49 ± 0.09 ^b^
	Feruloylcaffeoyltartaric acid 1	0.11 ± 0.01	0.34 ± 0.05 ^a^	0.11 ± 0.01 ^b^
	Feruloylcaffeoyltartaric acid 2	0.08 ± 0.005	0.24 ± 0.01 ^a^	0.08 ± 0.02 ^b^
Pomegranate peel	Punicalin ^a^	38.05 ± 7.83	14.69 ± 0.50 ^a^	11.66 ± 1.25 ^a,b^
	Punicalagin isomers ^a^	67.29 ± 1.59	68.63 ± 4.86	63.66 ± 3.64
	Punigluconin	4.13 ± 1.66	3.04 ± 1.36	2.74 ± 0.96
	Pedunculagin II	nd	3.36 ± 0.18	2.96 ± 0.39
	Galloyl-HHDP-hexose	2.06 ± 0.67	3.18 ± 0.33	3.74 ± 0.80
	Ellagic acid-hex	2.76 ± 0.32	5.88 ± 0.40 ^a^	5.83 ± 0.19 ^a^
	Gallagic acid	nd	1.05 ± 0.08	1.09 ± 0.12
	Granatin-B	0.51 ± 0.12	1.36 ± 0.09 ^a^	1.57 ± 0.25 ^a^
	Ellagic acid-pentose	nd	2.96 ± 0.46	3.41 ± 0.12
	Ellagic acid ^a^	9.58 ± 0.65	14.98 ± 0.47 ^a^	15.75 ± 0.38 ^a^

Values are expressed as mg/g extract. ^a^ Significant difference (*p* < 0.05) compared with non-fermented beverages. ^b^ Significant difference (*p* < 0.05) between beverages fermented with Strain A and fermented with Strain B. * Identified and quantified with their authentical standard.

**Table 3:** Antioxidant activities of lacto-fermented and non-fermented infusions.

	DPPH Activity (µmol eq Trolox/g of Extract)	FRAP Activity (µmol eq Trolox/g of Extract)	Xanthine Oxidase (XO) IC50 (mg/mL)
**Thyme** Non-fermented	92.87	439.16	1.45
Fermented A	323.93 ^a^	519.90	0.98 ^c^
Fermented B	306.46 ^a^	507.33	0.97 ^c^
**Rosemary** Non-fermented	100.26	203.63	2.11
Fermented A	347.92 ^a^	401.86 ^b^	1.35 ^c^
Fermented B	355.41 ^a^	367.54 ^b^	1.36 ^c^
**Echinacea** Non-fermented	262.95	383.30	0.32
Fermented A	439.38 ^a^	642.82 ^b^	0.35
Fermented B	445.41 ^a^	644.37 ^b^	0.37
**Pomegranate** **peel** Non-fermented	1104.25	1373.50	0.44
Fermented A	1233.28	1142.97	0.42
Fermented B	1060.01	1199.00	0.34

Effect of the lactic acid fermentations on the antioxidant activity (two-way ANOVA). ^a^ DPPH fermented (A or B) vs. non-fermented: *p* = 0.0159; fermented A vs. fermented B: *p* > 0.05. ^b^ FRAP fermented (A or B) vs. unfermented: *p* = 0.0357; fermented A vs. fermented B: *p* > 0.05. ^c^ XO fermented (A or B) vs. non-fermented: *p* = 0.0494; fermented A vs. fermented B: *p* > 0.05.

## Data Availability

All data included in this study are available upon request by contacting the corresponding authors.

## Supplementary Materials

The following supporting information can be downloaded at: https://www.mdpi.com/article/10.3390/antiox13050562/s1, Table S1: pH values of lacto-fermented herbal teas; Table S2: Content of phenolics of the different plant material used in this study; Table S3: Pearson correlations (r value) and p values in thyme infusions between the total phenolic content (TPC) and individual (poly)phenolics detected with the three pro-inflammatory markers (IL-6, IL-8, and PGE2) and antioxidant activities measured by DPPH, FRAP, and XO inhibition; Table S4: Pearson correlations (r value) and *p* values in rosemary infusions between the total phenolic content (TPC) and individual (poly)phenolics detected with the three pro-inflammatory markers (IL-6, IL-8, and PGE2) and antioxidant activities measured by DPPH, FRAP, and XO inhibition; Table S5: Pearson correlations (r value) and *p* values in echinacea infusions between the total phenolic content (TPC) and individual (poly)phenolics detected with the three pro-inflammatory markers (IL-6, IL-8, and PGE2) and antioxidant activities measured by DPPH, FRAP, and XO inhibition; Table S6: Pearson correlations (r value) and *p* values in pomegranate peel infusions between the total phenolic content (TPC) and individual (poly)phenolics detected with the three pro-inflammatory markers (IL-6, IL-8, and PGE2) and antioxidant activities measured by DPPH, FRAP, and XO inhibition.
