# Supplementary material for: Impact of Lactic Acid Bacteria Fermentation on (Poly)Phenolic Profile and In Vitro Antioxidant and Anti-Inflammatory Properties of Herbal Infusions
Source: Antioxidants (Basel). 2024 May 2;13(5):562. doi: 10.3390/antiox13050562 (PMC11117909; doi:10.3390/antiox13050562)
Supplement: Supplementary file 1 [file antioxidants-13-00562-s001.zip › antioxidants-2991088-supplementary.pdf]

Article

Impact of lactıc acid bacteria fermentation on (poly)phenolic profile and *ın vıtro* antioxidant and anti-inflammatory propertıes of novel herbal infusions

Tarik Ozturk[1‡], María Ángeles Ávila-Gálvez[2‡], Sylvie Mercier[3‡], Fernando Vallejo[2], Alexis Bred[3], Didier Fraisse[3], Christine Morand[3], Ebru Pelvan[1], Laurent-Emmanuel Monfoulet[3*] and Antonio González-Sarrías[2*]

Supplementary Materials

Supplementary Table S1. pH values of lacto-fermented herbal teas.

Plant Material	Basas (BF)	Strain A	Strain B
Thyme	6.52±0.02	4.23±0.02	4.21±0.01
Pomegranate Peel	5.22±0.02	3.85±0.01	3.71±0.02
Rosemary	6.81±-0.03	4.15±0.02	4.03±0.03
Echinacea	5.38±0.03	3.55±0.01	3.50±0.01

The data expressed as average ± SD (n=3), BF, before fermentation.

Supplementary Table S2. Content of phenolics in the various plant material used in this study

Plant material	RT	m/z⁻	MS/MS	Phenolic	mg/g extract
Thyme	12.69	447	285/267	Luteolin glucoside	16.49±1.97
	12.87	461	355/285	Chrysoeriol glucoside	109±1.00
	14.30	287	259/201/243	Eriodictyol	75.46±0.29
	14.77	359	223/197/161	Rosmarinic acid[a]	35.14±0.21
	14.93	301	255/239	Quercetin[a]	6.49±0.02
	15.64	537	313/295	Salvianolic acid A	4.74±0.47
Rosemary	13.29	477	462/315/300	Isorhamnetin-3-*O*-glucoside	25.10±0.24
	14.53	461	341/299/283	Hispidulin-7-*O*-glucoside	10.37±0.12
	14.78	359	223/197/161	Rosmarinic acid[a]	19.29±0.14
	14.92	609	463/301	Hesperidin[a]	12.94±0.14
	16.20	503	443/399/285	Luteo-lin-3-acetyl-*O*-glucuronide	9.09±0.07
	16.46	447	443/399/285	Luteolin glucoside	25.33±4.75
	16.81	623	477/315/300	Isorhamnetin-3-*O*-rutinoside	14.10±0.09
	22.02	345	301/283/259	Rosmanol peak1	0.87±0.01
	22.76	345	301/283/258	Rosmanol peak 2	1.61±0.01
	27.10	331	286	Carnosic acid	1.83±0.06
Echinacea	6.98	311	197/149	Caftaric acid	12.29±0.14
	8.22	353	311/191	Chlorogenic acid	1.20±0.13
	9.21	353	312/191/179	Neochlorogenic acid	1.15±0.08
	10.09	179	157/135	Caffeic acid[a]	0.84±0.06
	12.66	473	311/293/149	Chicoric acid[a]	49.11±0.52
	14.68	487	325/293/285	Feruloylcaffeoyltartaric acid 1	2.34±0.29
	14.93	487	325/293/179	Feruloylcaffeoyltartaric acid 2	1.48±0.04
Pomegranate peel	4.5	781	721/601/575	Punicalin[a]	17.36±2.61
	6.89/8.24	1083	781/721/601	Punicalagin isomers[a]	80.53±8.19
	8.66	799	479/391/301	Ellagic acid derivative	3.04±0.38
	9.24	801	649/347/301	Punigluconin	2.55±0.11
	9.56	785	633/483/301	Pedunculagin II	2.06±0.25
	10.19	633	463/301/275	Galloyl-HHDP-hexose	4.87±0.87
	10.50	463	301	Ellagic acid-hex	17.33±1.67
	10.99	601	299/271	Gallagic acid	15.80±3.21
	11.39	951	915/613/445	Granatin-B	1.78±0.37
	11.92	433	388/313/301	Ellagic acid-pentose	1.54±0.08
	12.20	447	301/257/229	Ellagic acid-deoxyhexose	6.60±0.23
	12.40	301	284/257/229	Ellagic acid[a]	59.01±5.84

[a]Identified and quantified with their authentical standard. RT: retention time.

Supplementary Table S3. Pearson correlations (r value) and *p* values in thyme infusions between the total phenolic content (TPC) and individual (poly)phenolics detected with the three pro-inflammatory markers (IL-6, IL-8 and PGE2) and antioxidant activities measured by DPPH, FRAP and XO inhibition.

Phenolic	IL-6 r value	IL-6 *p* value	IL-8 r value	IL-8 *p* value	PGE_2 r value	PGE_2 *p* value	DPPH r value	DPPH *p* value	FRAP r value	FRAP r value	XO r value	XO *p* value
**TPC**	-0.972	0.001	-0.973	0.001	-0.972	0.001	0.954	0.003	-0.148	0.780	-0.976	0.000
**Luteolin glucoside**	-0.992	0.926	-0.992	0.926	-0.992	0.927	0.998	0.041	-0.488	0.326	-0.990	0.000
**Chrysoeriol glucoside**	-0.945	0.004	-0.945	0.004	-0.945	0.004	0.921	0.009	-0.0519	0.922	-0.951	0.003
**Eriodictyol**	-0.937	0.006	-0.937	0.006	-0.937	0.006	0.959	0.002	-0.674	0.142	-0.931	0.007
**Rosmarinic acid**	0.108	0.837	0.109	0.837	0.109	0.837	-0.176	0.738	0.962	0.002	0.091	0.864
**Quercetin**	-0.939	0.005	-0.939	0.005	-0.939	0.005	0.961	0.002	-0.670	0.145	-0.933	0.006

Supplementary Table S4. Pearson correlations (r value) and *p* values in rosemary infusions between the total phenolic content (TPC) and individual (poly)phenolics detected with the three pro-inflammatory markers (IL-6, IL-8 and PGE2) and antioxidant activities measured by DPPH, FRAP and XO inhibition.

Phenolic	IL-6 r value	IL-6 *p* value	IL-8 r value	IL-8 *p* value	PGE_2 r value	PGE_2 *p* value	DPPH r value	DPPH *p* value	FRAP r value	FRAP r value	XO r value	XO *p* value
**TPC**	0.073	0.890	0.073	0.890	0.073	0.890	-0.099	0.852	0.089	0.866	0.061	0.907
**Isorhamnetin-3-glucoside**	-0.536	0.273	-0.535	0.273	-0.535	0.273	0.513	0.297	0.665	0.149	-0.545	0.263
**Hispidulin-7-O-glucoside**	-0.978	0.000	-0.978	0.000	-0.978	0.000	0.973	0.001	0.999	0.0170	-0.981	0.000
**Rosmarinic acid***	-0.364	0.478	-0.364	0.478	-0.364	0.478	0.388	0.447	0.208	0.692	-0.353	0.492
**Hesperidin***	-0.929	0.007	-0.929	0.007	-0.929	0.007	0.919	0.009	0.977	0.000	-0.933	0.006
**Luteolin-3-acetyl-O-glucuronide**	-0.995	0.428	-0.995	0.428	-0.995	0.428	0.997	0.136	0.965	0.002	-0.993	0.065
**Luteolin glucoside**	-0.935	0.006	-0.935	0.006	-0.935	0.006	0.926	0.008	0.980	0.000	-0.939	0.005
**Isorhamnetin-3-*O*-rutinoside**	-0.969	0.001	-0.969	0.001	-0.969	0.001	0.962	0.002	0.996	0.202	-0.972	0.001
**Rosmanol peak1**	-0.939	0.005	-0.939	0.005	-0.939	0.005	0.930	0.007	0.982	0.000	-0.943	0.005
**Rosmanol peak 2**	-0.958	0.003	-0.958	0.003	-0.9580	0.003	0.950	0.004	0.992	0.000	-0.961	0.002

Supplementary Table S5. Pearson correlations (r value) and *p* values in echinace infusions between the total phenolic content (TPC) and individual (poly)phenolics detected with the three pro-inflammatory markers (IL-6, IL-8 and PGE2) and antioxidant activities measured by DPPH, FRAP and XO inhibition.

**Phenolic**	**IL-6**	**IL-6**	**IL-8**	**IL-8**	**PGE_2**	**PGE_2**	**DPPH**	**DPPH**	**FRAP**	**FRAP**	**XO**	**XO**
	**r value**	***p* value**	**r value**	***p* value**	**r value**	***p* value**	**r value**	***p* value**	**r value**	**r value**	**r value**	***p* value**
**TPC**	-0.999	0.026	-0.999	0.026	-0.999	0.026	0.999	0.000	0.999	0.002	0.937	0.006
**Caftaric acid**	-0.715	0.110	-0.715	0.110	-0.715	0.110	0.695	0.125	0.714	0.111	0.379	0.459
**Chlorogenic acid**	-0.629	0.181	-0.629	0.181	-0.629	0.181	0.651	0.161	0.630	0.180	0.886	0.019
**Neochlorogenic acid**	-0.616	0.193	-0.616	0.193	-0.616	0.193	0.592	0.215	0.614	0.195	0.251	0.630
**Caffeic acid**	-0.001	0.999	-0.001	0.999	-0.001	0.999	0.029	0.966	0.002	0.997	0.397	0.435
**Chicoric acid**	-0.476	0.340	-0.476	0.340	-0.476	0.340	0.450	0.371	0.474	0.343	0.087	0.870
**Feruloylcaffeoyltartaric acid 1**	-0.500	0.312	-0.500	0.312	-0.500	0.312	0.475	0.341	0.499	0.315	0.115	0.829
**Feruloylcaffeoyltartaric acid 2**	-0.500	0.312	-0.500	0.312	-0.500	0.312	0.475	0.341	0.499	0.315	0.115	0.829

Supplementary Table S6. Pearson correlations (r value) and *p* values in pomegranate peel infusions between the total phenolic content (TPC) and individual (poly)phenolics detected with the three pro-inflammatory markers (IL-6, IL-8 and PGE2) and antioxidant activities measured by DPPH, FRAP and XO inhibition.

Phenolic	IL-6 r value	IL-6 *p* value	IL-8 r value	IL-8 *p* value	PGE_2 r value	PGE_2 *p* value	DPPH r value	DPPH *p* value	FRAP r value	FRAP r value	XO r value	XO *p* value
**TPC**	-0.998	0.057	-0.998	0.057	-0.998	0.006	0.211	0.688	0.352	0.493	-0.700	0.121
**Punicalin**	0.995	0.456	0.994	0.456	0.995	0.456	-0.170	0.748	-0.312	0.547	0.730	0.0993
**Punicalagin isomers**	0.257	0.623	0.257	0.623	0.257	0.623	0.860	0.028	0.776	0.0696	0.899	0.015
**Punigluconin**	0.979	0.000	0.979	0.000	0.979	0.000	-0.069	0.897	-0.214	0.684	0.796	0.059
**Pedunculagin II**	-0.991	0.000	-0.991	0.000	-0.991	0.000	0.398	0.434	0.528	0.281	-0.548	0.261
**Galloyl-HHDP-hexose**	-0.945	0.004	-0.945	0.004	-0.945	0.0045	-0.058	0.913	0.089	0.867	-0.866	0.026
**Ellagic acid-hex**	-0.999	0.000	-0.999	0.000	-0.999	0.000	0.285	0.584	0.423	0.404	-0.644	0.167
**Gallagic acid**	-0.999	0.001	-0.999	0.001	-0.999	0.010	0.233	0.657	0.373	0.467	-0.684	0.133
**Granatin-B**	-0.982	0.000	-0.982	0.000	-0.982	0.000	0.087	0.870	0.232	0.658	-0.784	0.065
**Ellagic acid-pentose**	-0.989	0.000	-0.989	0.000	-0.989	0.000	0.126	0.812	0.267	0.605	-0.760	0.080
**Ellagic acid**	-0.993	0.647	-0.993	0.647	-0.993	0.648	0.160	0.762	0.303	0.560	-0.737	0.095
